# GCOA-Net: a graph-regularized cross-omics attention network for interpretable breast cancer molecular subtype classification

**DOI:** 10.3389/fmed.2026.1790437

**Published:** 2026-04-10

**Authors:** Chen Li, Zhen Zhang, Chun Zhang

**Affiliations:** 1 Department of Breast Surgery, Peking University International Hospital, Beijing, China; 2 Department of Anesthesiology, Peking University First Hospital, Beijing, China

**Keywords:** breast cancer, calibration, graph neural networks, interpretability, molecular subtyping, multi-omics integration

## Abstract

**Introduction:**

Accurate intrinsic molecular subtyping is essential for precision management of breast cancer, yet multi-omics integration remains challenging due to high dimensionality, structured cross-omics dependencies, and the need for clinically interpretable and reliable predictions.

**Methods:**

We propose GCOA-Net, a graph-regularized cross-omics attention network that integrates transcriptomics, promoter-proximal DNA methylation, and miRNA expression. A biologically grounded heterogeneous graph connects CpG clusters to promoter-associated genes and miRNAs to their target genes. A relation-aware GNN encoder performs cross-omics message passing, while omics-specific and modality-level attention modules provide multi-level interpretability. We trained and evaluated models on TCGA-BRCA with repeated stratified five-fold cross-validation, benchmarking against classical early-fusion classifiers, integration frameworks, and deep multi-omics baselines. We additionally assessed ablations, subtype-specific explanations, robustness to missing modalities, calibration, and selective prediction.

**Results:**

GCOA-Net achieved the best overall performance (Acc = 0.912, Macro-F1 = 0.852, AUROC = 0.965) and improved calibration (ECE = 0.031) compared with baselines. Ablation analyses showed that biologically grounded cross-omics connectivity and graph regularization were key contributors, with degree-preserving edge randomization producing the largest performance drop. Attribution analyses identified subtype-consistent cross-omics biomarkers and compact explanatory subnetworks (e.g., ERBB2-centered regulation for HER2-enriched tumors). Under missing-modality scenarios, GCOA-Net degraded more gracefully and maintained better confidence reliability; selective prediction yielded a more favorable coverage–risk trade-off.

**Conclusion:**

Heterogeneous cross-omics graph modeling with graph regularization enables more accurate, robust, and interpretable breast cancer subtype classification, and provides a confidence-aware framework for molecular stratification that warrants further validation in independent multi-omics cohorts.

## 1 Introduction

Breast cancer is a biologically heterogeneous disease in which molecular subtypes capture clinically meaningful differences in prognosis and treatment response. Gene-expression profiling studies established intrinsic subtypes (e.g., Luminal A/B, HER2-enriched, Basal-like, Normal-like) with distinct clinical trajectories, providing a molecular taxonomy beyond histopathology alone (1, 2). Building on these discoveries, the PAM50 classifier operationalized intrinsic subtyping into a reproducible gene signature that has been widely adopted in translational research and clinical decision support (e.g., risk stratification and therapy selection) (3). Accurate and reliable subtype assignment therefore remains a central task for precision oncology, particularly for cases that present borderline phenotypes or ambiguous biomarker patterns.

Large-scale consortia have enabled systematic multi-omics characterization of breast tumors. The TCGA Breast Invasive Carcinoma (TCGA-BRCA) project profiled tumors across complementary molecular layers (including transcriptomics, DNA methylation, and microRNA regulation), revealing that subtype-defining programs are reflected across transcriptional, epigenetic, and post-transcriptional mechanisms (4). These data motivate integrative models that can jointly exploit cross-omics signals to improve subtype discrimination and to surface mechanistic hypotheses that are actionable for downstream validation. However, multi-omics learning is challenging: (i) features are high-dimensional and modality-specific noise is substantial; (ii) sample sizes are modest relative to dimensionality; (iii) cross-omics dependencies are structured (e.g., promoter methylation influencing gene activity; miRNAs regulating target genes), yet are often treated as unstructured correlations; and (iv) clinical translation requires not only higher accuracy but also calibrated probabilities and interpretable evidence to support risk-aware deployment.

A broad family of integration methods has been developed to address these issues. Classical early-fusion approaches concatenate features and apply standard classifiers, but can be dominated by high-variance modalities and may ignore inter-omics structure. Network- and latent-space integration methods, such as Similarity Network Fusion (SNF) and multivariate component models (e.g., mixOmics) aim to align samples across modalities while reducing dimensionality (5, 6). More recent probabilistic factorization frameworks (e.g., MOFA+) provide principled representations for multi-modal data and have become popular for extracting shared and modality-specific variation (7). Comprehensive reviews highlight that no single paradigm uniformly dominates across tasks, and that modern deep learning models are increasingly favored for capturing nonlinear cross-omics interactions, handling missingness, and enabling end-to-end prediction (8, 9). Recent graph-oriented perspectives further suggest that heterogeneous graph representations are becoming an increasingly important direction for integrated multi-omics analysis because they can encode cross-modal structure more explicitly than conventional fusion pipelines (10). Deep multi-omics learning has advanced rapidly, including graph-based methods that represent patients or features as graphs to propagate information and improve robustness. For example, MOGONET learns patient similarity graphs per modality and aggregates representations for classification, demonstrating the utility of graph inductive biases for multi-omics prediction (11). More recently, methods such as SUPREME, MVGNN, MOGAT, GAIN-BRCA, MO-GCAN, and MOFNet further explore graph-based or attention-based strategies for multi-omics integration and cancer subtype prediction (12–17). Despite these successes, two practical gaps remain for subtype-oriented clinical translation. First, many approaches build graphs from data-driven similarity rather than encoding biologically grounded cross-omics regulation (e.g., promoter regions linked to genes; miRNA–gene targeting), which limits mechanistic interpretability at the level of actionable molecular relationships. Second, predictive confidence is often under-examined: in clinical settings, probability estimates should be well-calibrated to support threshold-based referral, reflex testing, or selective prediction strategies (18). In parallel, interpretability has become a core requirement for high-stakes healthcare AI, where explanations should connect model evidence to domain-relevant entities and relationships (19).

Deep multi-omics learning has advanced rapidly, including graph-based methods that represent patients or features as graphs to propagate information and improve robustness. For example, MOGONET learns patient similarity graphs per modality and aggregates representations for classification, demonstrating the utility of graph inductive biases for multi-omics prediction (11). More recently, methods such as SUPREME further explore graph neural networks (GNNs) for multi-omics integration (12). Despite these successes, two practical gaps remain for subtype-oriented clinical translation. First, many approaches build graphs from data-driven similarity rather than encoding biologically grounded cross-omics regulation (e.g., promoter regions linked to genes; miRNA–gene targeting), which limits mechanistic interpretability at the level of actionable molecular relationships. Second, predictive confidence is often under-examined: in clinical settings, probability estimates should be well-calibrated to support threshold-based referral, reflex testing, or selective prediction strategies (18). In parallel, interpretability has become a core requirement for high-stakes healthcare AI, where explanations should connect model evidence to domain-relevant entities and relationships (19).

**Figure 1:**
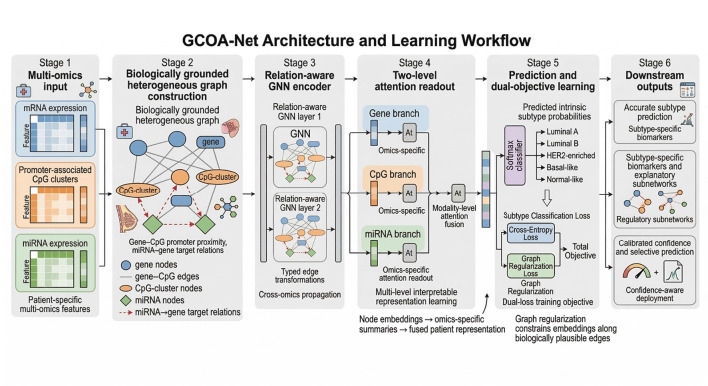
Overview of the GCOA-Net framework.

In this work, we propose a Graph-regularized Cross-Omics Attention Network (GCOA-Net) for breast cancer intrinsic subtype classification that is explicitly designed for clinical interpretability and deployment-oriented reliability. As summarized in Figure 1, GCOA-Net first maps patient-specific multi-omics measurements (mRNA, promoter-proximal CpG clusters, and miRNAs) onto a biologically grounded heterogeneous molecular graph, where edges encode mechanistic priors from gene–CpG proximity and curated miRNA–gene targeting knowledge (20). A relation-aware GNN encoder then propagates signals across omics layers to capture structured cross-omics dependencies (21), while a graph regularization term encourages representation consistency along biologically plausible edges, improving stability under repeated cross-validation and partial modality loss (22). Crucially, we couple this architecture with attribution-based interpretability, decomposing evidence into salient nodes and cross-omics edges and extracting sparse, subtype-specific explanatory subnetworks that highlight candidate regulatory programs. We evaluate GCOA-Net on TCGA-BRCA under repeated stratified cross-validation, benchmarking against classical, integration, and deep multi-omics baselines, and further assess robustness to missing modalities and calibration to facilitate confidence-aware downstream use.

The main contributions of this study are as follows. First, we formulate breast cancer intrinsic subtype classification as a multi-omics prediction problem that emphasizes not only discrimination performance but also biological interpretability and confidence-aware reliability, thereby aligning the task more closely with the needs of subtype-oriented translational analysis. Second, we propose GCOA-Net, a heterogeneous cross-omics architecture that represents genes, promoter-associated CpG clusters, and miRNAs within a biologically grounded molecular graph, and combines relation-aware message passing with omics-specific and modality-level attention to capture structured cross-omics dependencies. Third, we introduce a graph-regularized learning strategy that encourages representation consistency along biologically plausible cross-omics edges, improving robustness and stabilizing feature propagation beyond attention-based fusion alone. Fourth, we provide a comparatively comprehensive evaluation framework that includes not only benchmark discrimination performance, but also subtype-wise error analysis, ablation experiments, attribution-based interpretability, missing-modality robustness, calibration, selective prediction, corrected repeated cross-validation inference, and site-stratified internal generalization. Compared with prior multi-omics subtype prediction studies, these contributions differ from existing approaches in several important ways. Relative to classical early-fusion classifiers and latent-component integration frameworks such as SNF and DIABLO, GCOA-Net does not treat cross-omics dependence as either simple feature concatenation or sample-level similarity alignment, but instead models explicit molecular relations among genes, promoter-associated CpG clusters, and miRNAs. Relative to patient-similarity graph methods such as MOGONET and SUPREME, the proposed graph is constructed at the molecular-entity level rather than at the patient level, which improves the biological specificity and interpretability of message passing. Relative to attention-based breast cancer subtype models such as moBRCA-net, our method adds a graph-regularized heterogeneous relational layer that constrains cross-omics information flow using biologically grounded priors. Finally, beyond architectural novelty, the present study extends recent work by evaluating not only discrimination performance, but also subtype-specific explanatory subnetworks, calibration, selective prediction, corrected repeated cross-validation inference, and site-stratified internal generalization.

## 2 Materials and methods

### 2.1 Data source, cohort, and labels

**(1) TCGA cohort and molecular subtypes:** we used the same TCGA Breast Invasive Carcinoma (TCGA-BRCA) multi-omics cohort and PAM50 intrinsic subtype labels as in moBRCA-net (23). TCGA provides de-identified, publicly available genomic profiles for large cancer cohorts (4, 24). Intrinsic subtypes were defined by the PAM50 classifier (Luminal A, Luminal B, HER2-enriched, Basal-like, Normal-like) (3). After harmonizing sample identifiers across modalities and excluding cases with missing subtype labels, the final cohort contained *N* = 1, 059 patients.

**(2) Omics modalities:** we modeled three modalities per patient: (i) mRNA expression at the gene level, (ii) promoter-proximal DNA methylation summarized as CpG-cluster features, and (iii) miRNA expression. Following the standard feature harmonization convention, the retained feature spaces contained 20, 400 genes, 19, 977 promoter CpG clusters, and 1, 597 miRNAs.

### 2.2 Preprocessing and fold-Safe standardization

**(1) Within-fold preprocessing:** to prevent information leakage, all transformations that depend on data moments (e.g., standardization) were computed *within each training fold only* and then applied to the corresponding validation/test splits. Let ${\text{x}}_{i}^{\left(t\right)}\in {\mathbb{R}}^{d_{t}}$ denote patient *i*'s raw feature vector for modality *t* ∈ {*g, c, m*} (gene, CpG, miRNA). We applied z-standardization using training-fold statistics:


$$\begin{array}{l} {\tilde{\text{x}}}_{i}^{\left(t\right)}=\frac{{\text{x}}_{i}^{\left(t\right)}-\mu_{\text{train}}^{\left(t\right)}}{\sigma_{\text{train}}^{\left(t\right)}+\epsilon }, \end{array}$$ (1)

where $\mu_{\text{train}}^{\left(t\right)}$ and $\sigma_{\text{train}}^{\left(t\right)}$ in Equation 1 are computed over the training split and ϵ is a small constant for numerical stability.

**(2) Missingness policy for robustness tests:** in the missing-modality analyses, we simulated test-time absence by masking the corresponding standardized inputs without retraining model parameters.

### 2.3 Biologically grounded heterogeneous cross-omics graph construction

**Figure 2:**
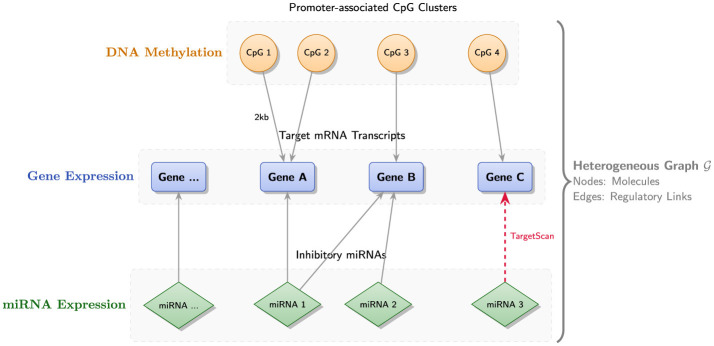
Schematic illustration of heterogeneous graph construction in GCOA-Net. The graph G integrates three layers of omics data: DNA methylation **(top)**, gene expression **(middle)**, and miRNA expression **(bottom**). Nodes represent biological entities, including promoter-associated CpG clusters, genes, and miRNAs. Edges encode biologically grounded regulatory relations: (1) Gene–CpG links connect promoter-associated CpG clusters to genes when the cluster falls within the strand-aware promoter span defined from 2,000 bp upstream to 500 bp downstream of the annotated transcription start site; (2) miRNA → gene links connect miRNAs to retained target genes based on TargetScan Human version 8.0 conserved target-site relationships after confidence filtering.

We summarize the key annotation choices, filtering rules, identifier harmonization steps, and graph assembly counts (illustrated in Figure 2).

**(1) Graph definition:** we constructed a fixed heterogeneous graph G = (V, E) with typed node sets V = V_*g*_ ∪ V_*c*_ ∪ V_*m*_, representing genes, promoter-associated CpG clusters, and miRNAs, respectively. Cross-omics edges encoded biologically grounded relations:


$$\begin{array}{l} E=E_{g\text{–}c}\;\cup \;E_{m\to g}, \end{array}$$ (2)

$$\begin{array}{ll} E_{g\text{–}c} & :=\left\{\left(c,g\right)|c\;\text{maps to the promoter region of }g\right\}, \end{array}$$ (3)

$$\begin{array}{ll} E_{m\to g} & :=\left\{\left(m,g\right)|g\;\text{is a retained target of }m\right\}. \end{array}$$ (4)

All genomic coordinates used to define the relations in Equations 2–4 were represented on the GRCh38 reference build, which is the native coordinate system of the TCGA GDC data used in this study. Gene annotation was based on GENCODE v36. For genes with multiple annotated isoforms, we used the union of transcription start sites to define a gene-level promoter span. Promoter regions were then defined directionally, according to gene strand, as the interval from 2,000 bp upstream to 500 bp downstream of each annotated transcription start site. A CpG cluster was linked to a gene if its genomic position fell within the promoter span of that gene. Because promoter intervals may overlap across genes, the resulting gene–CpG relation was allowed to be many-to-many.

DNA methylation features were derived from Illumina HumanMethylation450 probe-level data. Probe coordinates were taken from the hg38-mapped annotation provided by the GDC. To reduce probe-level noise and obtain regionally more stable methylation features, probes lying within 500 bp of one another were merged into the same CpG cluster. Only clusters containing at least two probes were retained, and cluster-level methylation was summarized as the arithmetic mean of the beta values of the constituent probes. Before aggregation, probes with more than 10% missing samples were removed. This procedure yielded 19,977 promoter-related CpG clusters for downstream graph construction.

miRNA→gene edges were obtained from TargetScan Human version 8.0 (Whitehead Institute for Biomedical Research, Cambridge, MA, USA) (20). We retained only conserved target-site relationships and applied a confidence filter based on the cumulative weighted context++ score, keeping interactions with score ≤ −0.2. To align external annotations with the retained TCGA feature space, gene identifiers were harmonized using biomaRt, and miRNA features were standardized to mature miRNA names based on miRBase v22. Entities not present in the preprocessed TCGA expression or miRNA matrices were excluded. When multiple candidate features mapped to the same retained TCGA feature identifier, we retained the instance with the largest cross-sample variance. Repeated mappings that yielded the same final node pair were collapsed into a unique edge. A structured summary of the genomic reference build, promoter definition, CpG clustering rule, aggregation strategy, miRNA target filtering, and identifier harmonization procedure is provided in Supplementary Table S1.

After harmonization and filtering, the final graph contained 20,400 gene nodes, 19,977 CpG-cluster nodes, and 1,597 miRNA nodes. The graph included 34,228 gene–CpG edges and 348,387 miRNA→gene edges, giving a total of |V| = 41, 974 nodes and |E| = 382, 615 edges. This prior graph was constructed once before cross-validation and then used unchanged across all repeated training and evaluation runs. Supplementary Table S2 reports the node and edge counts at each major stage of graph assembly, from raw TCGA and external database features to the final heterogeneous graph used for model training.

**(2) Patient-specific graph signals:** for each patient *i*, node features are the modality-specific measurements placed on the corresponding node types, yielding an initial graph signal:


$$\begin{array}{l} {\text{H}}_{i}^{\left(0\right)}={\left\{{\text{h}}_{v,i}^{\left(0\right)}\in {\mathbb{R}}^{1}\right\}}_{v\in V}, \end{array}$$ (5)

where ${\text{h}}_{v,i}^{\left(0\right)}$ in Equation 5 is the standardized value for the molecular feature represented by node *v* for patient *i*. This formulation allows message passing to combine patient-specific signals along biologically grounded cross-omics edges.

### 2.4 GCOA-net architecture

GCOA-Net couples (i) a relation-aware heterogeneous GNN encoder to learn cross-omics dependencies and (ii) an attention-based readout/fusion module to produce a patient-level representation for subtype classification.

**(1) Relation-aware heterogeneous message passing:** let R = {*g*–*c, m* → *g*} denote relation types. We adopt an R-GCN-style update (21) with type-specific parameters. For node *v*, layer ℓ → ℓ + 1 updates:


$$\begin{array}{l} {\text{h}}_{v,i}^{\left(\ell +1\right)}=\sigma \left({\text{W}}_{0}^{\left(\ell \right)}{\text{h}}_{v,i}^{\left(\ell \right)}+\underset{r\in R}{\sum }\underset{u\in N_{r}\left(v\right)}{\sum }\frac{1}{c_{v,r}}{\text{W}}_{r}^{\left(\ell \right)}{\text{h}}_{u,i}^{\left(\ell \right)}\right), \end{array}$$ (6)

where N_*r*_(*v*) in Equation 6 is the neighborhood of *v* under relation *r*, *c*_*v, r*_ is a normalization constant (e.g., |N_*r*_(*v*)|), σ(·) is a nonlinearity, and ${\text{W}}_{r}^{\left(\ell \right)}$ are learnable matrices. We stack *L* layers to obtain final node embeddings ${\text{h}}_{v,i}^{\left(L\right)}$ that integrate multi-omics evidence through cross-omics connectivity.

**(2) Omics-specific attentive readout and cross-omics fusion:** because subtype prediction is patient-level, we summarize node embeddings into modality-level representations and then fuse them with attention. For each node type *t* ∈ {*g, c, m*}, we compute an attention-weighted readout:


$$\begin{array}{l} \alpha_{v,i}^{\left(t\right)}=\frac{\exp\left({\text{a}}_{t}^{⊤}\tanh\left({\text{W}}_{t}{\text{h}}_{v,i}^{\left(L\right)}\right)\right)}{\sum_{v^{\prime }\in V_{t}}\exp\left({\text{a}}_{t}^{⊤}\tanh\left({\text{W}}_{t}{\text{h}}_{v^{\prime },i}^{\left(L\right)}\right)\right)}, \end{array}$$ (7)

$$\begin{array}{l} {\text{z}}_{i}^{\left(t\right)}=\underset{v\in V_{t}}{\sum }\alpha_{v,i}^{\left(t\right)}{\text{h}}_{v,i}^{\left(L\right)}. \end{array}$$ (8)

We then fuse the three modality vectors derived from Equations 7 and 8 using a second-stage attention:


$$\begin{array}{l} \beta_{i}^{\left(t\right)}=\text{softma}{\text{x}}_{t}({\text{q}}^{⊤}\tanh\left(\text{U}{\text{z}}_{i}^{\left(t\right)}\right)), \end{array}$$ (9)

$$\begin{array}{l} {\text{z}}_{i}=\underset{t\in \left\{g,c,m\right\}}{\sum }\beta_{i}^{\left(t\right)}{\text{z}}_{i}^{\left(t\right)}. \end{array}$$ (10)

Finally, subtype probabilities are computed by a softmax classifier:


$$\begin{array}{l} {\hat{\text{p}}}_{i}=\text{softmax}\left({\text{W}}_{o}{\text{z}}_{i}+{\text{b}}_{o}\right). \end{array}$$ (11)

This design yields (i) node-level attention for within-omics interpretability and (ii) modality-level attention for cross-omics contribution analysis.

### 2.5 Graph regularization objective

To encourage biologically coherent representations along known cross-omics relations, we add a Laplacian-style smoothness regularizer (manifold/graph regularization) (22) on the final-layer embeddings fused in Equations 9–11:


$$\begin{array}{l} L_{\text{gr}}=\underset{r\in R}{\sum }\underset{\left(u,v\right)\in E_{r}}{\sum }w_{uv}\|{\text{h}}_{u,i}^{\left(L\right)}-{\text{h}}_{v,i}^{\left(L\right)}{\|}_{2}^{2}, \end{array}$$ (12)

where *w*_*uv*_ is an optional edge weight (set to 1 in our experiments unless otherwise specified). Intuitively, Equation 12 penalizes large embedding discrepancies across biologically supported edges, stabilizing cross-omics message passing and improving calibration/robustness.

### 2.6 Training loss and optimization

**(1) Supervised objective:** given true subtype label *y*_*i*_ ∈ {1, …, *K*} (*K* = 5), we minimize the regularized cross-entropy:


$$\begin{array}{l} L=\underset{L_{\text{CE}}}{\underset{︸}{-\underset{i\in B}{\sum }\log{\hat{p}}_{i,y_{i}}}}+\lambda_{\text{gr}}\underset{\text{graph regularization}}{\underset{︸}{\underset{i\in B}{\sum }L_{\text{gr}}\left(i\right)}}+\lambda_{\text{wd}}\|\Theta {\|}_{2}^{2}, \end{array}$$ (13)

where B in Equation 13 is a minibatch and Θ denotes all trainable parameters.

**(2) Optimization and early stopping:** models were trained with Adam (25) and early stopping using a held-out validation split (10% of the training fold). All baselines and GCOA-Net used the same fold partitions and preprocessing rules for fair comparison. Implementation used PyTorch (26) with graph modules in PyTorch Geometric (27).

### 2.7 Baselines

We benchmarked against three baseline families: classical early-fusion machine learning methods, including SVM with RBF kernel (28), Random Forest (29), XGBoost (30), and Elastic Net logistic regression (31); integration frameworks, including SNF + SVM (5) and DIABLO from mixOmics (32); and deep multi-omics models, including a DeepMO-style late-fusion baseline (33), MOGONET (11), SUPREME (12), and moBRCA-net (23). When official public implementations were available, we used the original codebase with only task-specific data-interface adaptation where needed. For package-based baselines, we used established software implementations under their standard workflows. The DeepMO-style late-fusion baseline was implemented following the original methodological description. All baselines were evaluated using the same outer repeated stratified five-fold splits and the same fold-safe preprocessing pipeline as GCOA-Net. Hyperparameter selection, when applicable, was performed using only the training portion of each outer split. Supplementary Table S3 reports the implementation source, software environment, tuning strategy, key hyperparameter ranges, and stopping rule for each baseline and for GCOA-Net.

### 2.8 Evaluation protocol and metrics

**(1) Repeated stratified cross-validation:** all reported results were obtained with stratified five-fold cross-validation repeated 5 times using shared outer splits across all methods. For each run, one fold was used for testing, while the remaining folds were used for model fitting. This design ensured that baseline tuning and final evaluation were conducted under the same data-partitioning protocol.

**(2) Primary discrimination metrics:** we report Accuracy, Balanced Accuracy, Matthews Correlation Coefficient (MCC), Macro-F1, and Weighted-F1. For class *k*, precision *P*_*k*_, recall *R*_*k*_, and F1:


$$\begin{array}{l} F1_{k}=\frac{2P_{k}R_{k}}{P_{k}+R_{k}}. \end{array}$$ (14)

Macro-F1 and Weighted-F1 are:


$$\begin{array}{l} \text{Macro-F}1=\frac{1}{K}\overset{K}{\underset{k=1}{\sum }}F1_{k},\text{ W}\text{-F}1=\overset{K}{\underset{k=1}{\sum }}\frac{n_{k}}{N}F1_{k}, \end{array}$$ (15)

where *n*_*k*_ in Equation 15 is the support for class *k*, while precision, recall, and class-wise F1 are defined in Equation 14. AUROC and AUPRC were computed in a one-vs-rest manner and macro-averaged across classes.

**(3) Calibration metrics:** we evaluated calibration using Expected Calibration Error (ECE) (18, 34), Brier score (35), and Negative Log-Likelihood (NLL). For ECE, predictions are partitioned into *M* confidence bins ${\left\{B_{m}\right\}}_{m=1}^{M}$:


$$\begin{array}{l} \text{ECE}=\overset{M}{\underset{m=1}{\sum }}\frac{\left|B_{m}\right|}{N}|\text{acc}\left(B_{m}\right)-\text{conf}\left(B_{m}\right)|. \end{array}$$ (16)

Calibration error curves in Equation 16 (reliability diagrams) plot acc(*B*_*m*_) vs. conf(*B*_*m*_) across bins.

**(4) Statistical comparison of model performance:** for inferential comparison between GCOA-Net and competing methods, we used paired performance differences computed on the shared outer test folds of the repeated stratified five-fold cross-validation procedure. To account for the dependence induced by repeated cross-validation, statistical significance was assessed using the two-sided Nadeau–Bengio corrected repeated cross-validation test (36). In addition to corrected *P*-values, we report corrected 95% confidence intervals for the mean performance difference. The corrected inferential analysis was applied to the primary comparison metrics, including Accuracy, Macro-F1, AUROC, and AUPRC. For five-fold cross-validation, the correction used the corresponding test-to-train ratio of 1/4.

### 2.9 Ablation, interpretability, and deployment-oriented analyses

**(1) Ablation study:** to isolate the contributions of heterogeneous message passing and graph regularization, we evaluated structured ablations by removing individual components while keeping all other settings fixed.

**(2) XAI-based attributions and explanatory subnetworks:** to improve interpretability, we used established gradient-based Explainable AI (XAI) methods to quantify subtype-specific node and edge importance (37, 38). For a test sample *i* and predicted logit *s*_*i,k*_ for subtype *k*, node-level importance for node *v* was computed using saliency-style gradients with respect to the input feature (or final embedding), and was additionally summarized using an Integrated Gradients perspective to improve stability of attribution. The resulting node scores were aggregated as absolute importance values and averaged across out-of-fold test predictions. To score edges, we used differentiable edge gating: each edge *e* was assigned a continuous gate *g*_*e*_ (initialized to 1) multiplying its message contribution, and edge importance was derived from |∂*s*_*i, k*_/∂*g*_*e*_| or its integrated-gradient variant. Subtype-specific explanatory subnetworks were then extracted by ranking cross-omics edges by aggregated attribution magnitude and retaining the top 1% edges, followed by connected-component pruning to yield compact, interpretable graphs.

**(3) Robustness to missing modalities:** we simulated missing-modality scenarios at inference time by masking one modality, and additionally considered a severe-loss setting, while keeping the model parameters fixed. For GCOA-Net, the corresponding node-type features were set to zero after standardization and excluded from attention readout via a modality mask. To examine whether explicit missingness-aware training further improves robustness, we additionally trained a variant of GCOA-Net with training-time masking, in which an entire modality was randomly masked with probability 0.2 during model fitting. This variant was evaluated under the same inference-time missingness scenarios as the standard model.

**(4) Selective prediction with abstention:** to emulate safety-oriented deployment, we adopted a confidence-based reject option in which the model abstains when $\max_{k}{\hat{p}}_{i,k}<\tau$ (39, 40). Rather than choosing τ on the test data, we selected abstention thresholds on the internal validation split using pre-specified target coverage rates of 90%, 80%, and 70%. The resulting thresholds were then applied unchanged to the corresponding held-out test fold.

**(5) Site-stratified internal generalization:** to assess generalization under a stronger within-cohort distribution shift, we conducted a site-held-out evaluation using the five Tissue Source Site groups in TCGA-BRCA (BH, A2, AR, E2, and AC). In each run, one site was held out entirely for testing and the remaining selected sites were used for model fitting, including training-fold preprocessing, hyperparameter selection, and early stopping. We summarize performance by the mean metric across held-out sites and by worst-site performance, thereby evaluating both average transferability and the lower bound of cross-site robustness.

## 3 Results

### 3.1 Baseline models and comparative evaluation

To strictly benchmark the performance of our proposed **GCOA-Net**, we compared it against a comprehensive suite of computational methods, ranging from classical machine learning to state-of-the-art deep multi-omics frameworks. The baselines are categorized into three groups:

1. **Classical classifiers (early fusion):** support Vector Machines (SVM) (28), Random Forests (RF) (29), XGBoost (30), and Elastic Net Logistic Regression (EN-Logit) (31). These models were trained on the simple concatenation of all omics features.
2. **Omics-integration frameworks:** Similarity Network Fusion (SNF) (5) coupled with SVM, and DIABLO (32) (mixOmics), representing statistical latent space integration methods.
3. **Deep learning and GNNs:** DeepMO-style late fusion networks (33) and graph-based methods including MOGONET (11), SUPREME (12), and the direct competitor moBRCA-net (23).

Across the full benchmark suite, performance generally increased from classical early-fusion classifiers to deep multi-omics frameworks, with GCOA-Net achieving the strongest overall results (Table 1). Among classical early-fusion baselines, SVM (RBF) performed best (Acc = 0.866, Macro-F1 = 0.808, MCC = 0.808), whereas tree-based methods showed lower accuracy (e.g., RF: Acc = 0.825). Omics-integration frameworks (SNF + SVM and DIABLO) yielded competitive ranking-based metrics (e.g., DIABLO: AUROC = 0.929, AUPRC = 0.851), but their overall accuracy remained below the strongest deep models. Within deep learning and GNN baselines, moBRCA-net was the best-performing comparator (Acc = 0.891, Macro-F1 = 0.835, AUROC = 0.952), indicating that attention-based cross-omics fusion provides a strong starting point for multi-omics subtype prediction. GCOA-Net surpassed all baselines across every reported metric, reaching Acc = 0.912, Macro-F1 = 0.852, W-F1 = 0.910, AUROC = 0.965, AUPRC = 0.915, and MCC = 0.865 (Table 1).

**Table 1:** Comparative performance of GCOA-Net and baseline models on breast cancer subtype classification.

Model	Mechanism	Acc.	Macro-F1	W-F1	AUROC	AUPRC	MCC
Classical ML (early fusion)							
SVM (RBF)	Kernel method	0.866	0.808	0.861	0.903	0.812	0.808
Random Forest	Ensemble tree	0.825	0.758	0.819	0.910	0.821	0.758
XGBoost	Gradient boost	0.845	0.775	0.838	0.918	0.833	0.775
EN-Logit	Linear (Reg.)	0.852	0.784	0.849	0.913	0.826	0.784
Integration frameworks							
SNF + SVM	Similarity net	0.828	0.771	0.784	0.925	0.844	0.771
DIABLO	Latent components	0.835	0.781	0.792	0.929	0.851	0.781
Deep learning and GNNs							
DeepMO-style	Late fusion MLP	0.846	0.795	0.806	0.937	0.861	0.795
MOGONET	Patient graph	0.858	0.811	0.820	0.944	0.872	0.811
SUPREME	Patient graph	0.863	0.817	0.826	0.948	0.878	0.817
moBRCA-net	Attention fusion	0.891	0.835	0.887	0.952	0.885	0.831
**GCOA-Net**	**Mol. graph Reg**.	**0.912**	**0.852**	**0.910**	**0.965**	**0.915**	**0.865**

Values represent mean performance metrics across repeated five-fold cross-validation. Macro-F1 denotes the unweighted average of class-wise F1-scores, whereas W-F1 denotes the support-weighted F1-score. Bold values indicate the best result in each metric column across all methods.

**Figure 3:**
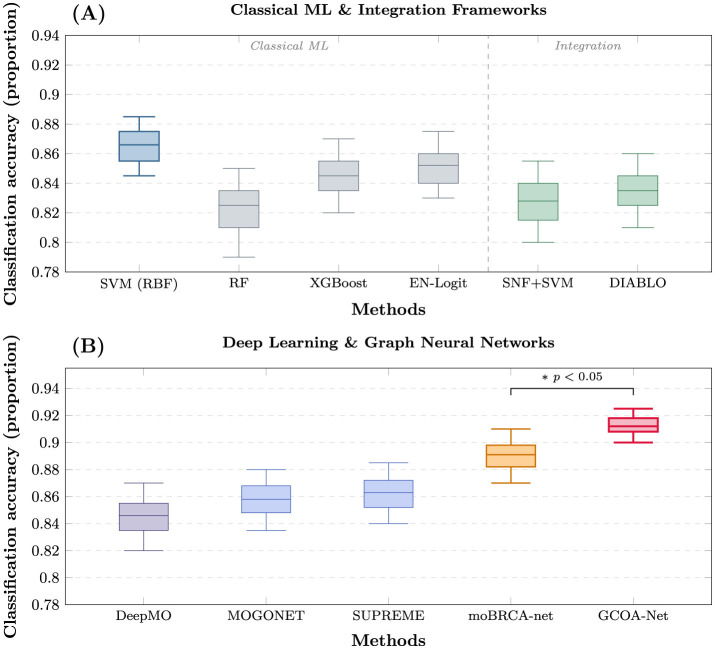
Comparative performance distribution across repeated five-fold cross-validation. The box plots illustrate the distribution of classification accuracy for **(A)** classical machine learning baselines and integration frameworks, and **(B)** deep learning and graph neural network approaches. The box represents the interquartile range, the horizontal line denotes the median, and whiskers extend to 1.5 × IQR. Statistical comparison between GCOA-Net and moBRCA-net was conducted using the two-sided Nadeau–Bengio corrected repeated cross-validation test. The symbol * indicates a statistically significant difference (*p* < 0.05).

Relative to moBRCA-net, GCOA-Net improved accuracy by +0.021 (0.912 vs. 0.891) and achieved consistent gains in Macro-F1 (+0.017), Weighted-F1 (+0.023), MCC (+0.034), AUROC (+0.013), and AUPRC (+0.030), suggesting that explicitly modeling biologically grounded cross-omics relations can enhance both overall discrimination and balanced multi-class performance. Corrected inferential comparison for Accuracy, Macro-F1, AUROC, and AUPRC is reported in Supplementary Table S4. Fold-level distributions further corroborate these findings (Figure 3). In Figure 3A, classical and integration methods exhibit broader variability and lower medians compared with the top deep models. In Figure 3B, GCOA-Net shows the highest median accuracy with a consistently strong distribution across repeated splits. Relative to moBRCA-net, the improvement remained statistically significant under the two-sided Nadeau–Bengio corrected repeated cross-validation test, with corrected 95% confidence intervals excluding zero for Accuracy, Macro-F1, AUROC, and AUPRC (Supplementary Table S4). The mean-metric comparison and the fold-level distributions indicate that the proposed graph-regularized heterogeneous modeling provides a consistent empirical advantage over both classical baselines and strong deep multi-omics comparators.

### 3.2 Subtype-wise performance and error patterns

**Figure 4:**
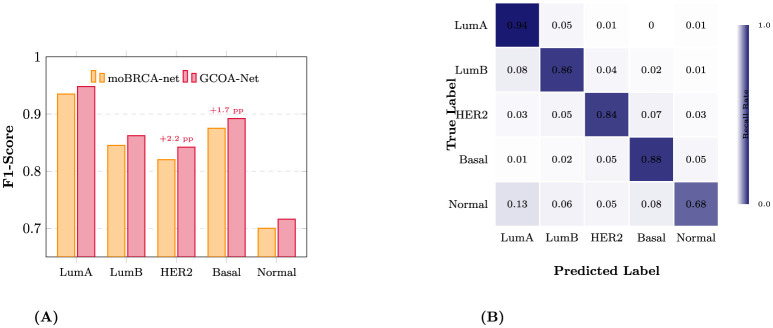
Subtype-specific performance and error analysis. **(A)** Subtype-wise F1-score comparison between the baseline moBRCA-net (orange) and the proposed GCOA-Net (red). **(B)** Row-normalized confusion matrix for GCOA-Net. The diagonal elements represent the recall for each subtype.

Table 2 shows that the overall improvement of GCOA-Net is driven by *consistent* subtype-wise gains rather than performance trade-offs across classes. Compared with the strong deep baseline moBRCA-net, GCOA-Net achieved higher F1-scores for all five PAM50 subtypes: Luminal A (0.935 → 0.948, +0.013), Luminal B (0.845 → 0.862, +0.017), HER2-enriched (0.820 → 0.842, +0.022), Basal-like (0.875 → 0.892, +0.017), and Normal-like (0.700 → 0.716, +0.016) (Figure 4A). These gains were jointly supported by improvements in both precision and recall. For example, precision increased for HER2-enriched from 0.810 to 0.850 (+0.040) and recall increased for Basal-like from 0.861 to 0.880 (+0.019), indicating that the model better identifies subtype-defining signals while reducing false positives/negatives.

**Table 2:** Subtype-wise performance comparison between moBRCA-net and GCOA-Net.

Subtype	Support (*n*)	Model	Precision	Recall	F1-score
Luminal A	542	moBRCA-net	0.944	0.927	0.935
		**GCOA-Net**	**0.956**	**0.940**	**0.948**
Luminal B	211	moBRCA-net	0.842	0.848	0.845
		**GCOA-Net**	**0.868**	**0.856**	**0.862**
HER2-enriched	118	moBRCA-net	0.810	0.833	0.820
		**GCOA-Net**	**0.850**	**0.835**	**0.842**
Basal-like	144	moBRCA-net	0.890	0.861	0.875
		**GCOA-Net**	**0.904**	**0.880**	**0.892**
Normal-like	44	moBRCA-net	0.737	0.667	0.700
		**GCOA-Net**	**0.760**	**0.676**	**0.716**

Within each subtype, bold values indicate the better result between moBRCA-net and GCOA-Net for that metric.

The normalized confusion matrix (Table 3; Figure 4B) further characterizes error structure. Diagonal entries (recall) were highest for Luminal A (0.940) and Basal-like (0.880), followed by Luminal B (0.856) and HER2-enriched (0.835), whereas Normal-like remained the most challenging subtype (0.676), likely reflecting its small support (*n* = 44) and its known proximity to luminal expression programs. Misclassifications largely occurred between biologically adjacent categories: Luminal A was most frequently predicted as Luminal B (0.045), and Luminal B was most frequently predicted as Luminal A (0.080). HER2-enriched showed residual confusion with Basal-like (0.065) and Luminal B (0.050), while Basal-like occasionally overlapped with HER2-enriched (0.050) and Normal-like (0.045). For Normal-like cases, the dominant confusion was toward Luminal A (0.130), with smaller spillover to Basal-like (0.084) and Luminal B (0.060). Overall, the confusion patterns suggest that remaining errors are concentrated in subtype pairs with overlapping molecular profiles, while GCOA-Net improves subtype discrimination broadly without introducing new systematic failure modes.

**Table 3:** Row-normalized confusion matrix for GCOA-Net.

	Predicted label				
True label	LumA	LumB	HER2	Basal	Normal
Luminal A	**0.940**	0.045	0.005	0.000	0.010
Luminal B	0.080	**0.856**	0.040	0.015	0.009
HER2-enriched	0.025	0.050	**0.835**	0.065	0.025
Basal-like	0.010	0.015	0.050	**0.880**	0.045
Normal-like	0.130	0.060	0.050	0.084	**0.676**

Diagonal elements represent the Recall (Sensitivity) for each subtype. Significant confusion is observed between Luminal A/B and Normal-like/Luminal A, reflecting biological similarities. Bold values indicate the largest value in each row of the row-normalized confusion matrix.

Overall, the confusion patterns suggest that remaining errors are concentrated in subtype pairs with overlapping molecular profiles, while GCOA-Net improves subtype discrimination broadly without introducing new systematic failure modes; clinically, the persistent Luminal A/B and Normal-like boundary ambiguity is commonly observed in TCGA-style profiling due to shared luminal programs and limited Normal-like representation, motivating the subsequent interpretability analyses to verify whether the model's high-confidence decisions are supported by subtype-consistent cross-omics markers and regulatory subnetworks.

### 3.3 Ablation study: effects of heterogeneous graph modeling and graph regularization

To quantify the contributions of (i) explicit cross-omics heterogeneous graph modeling and (ii) graph regularization, we conducted a structured ablation study under the same repeated stratified CV protocol used in Sections 3.1–3.2. Starting from the full GCOA-Net, we removed or perturbed one component at a time while keeping all other settings fixed (optimizer, early stopping, and within-fold preprocessing). The key ablations were: (1) *w/o graph regularization* (λ_gr_ = 0); (2) *w/o GNN message passing* (replacing the graph encoder with independent modality encoders and fusion-only attention); (3) *w/o miRNA* → *gene edges* (retaining only gene–CpG edges); (4) *w/o gene–CpG edges* (retaining only miRNA–gene edges); (5) *randomized cross-omics edges* (degree-preserving random rewiring of cross-omics edges), which tests whether gains depend on biologically grounded connectivity rather than generic graph smoothing.

Table 4 quantifies the contribution of each architectural and graph-design component. Removing graph regularization (λ_gr_ = 0) led to a consistent degradation across discrimination and calibration metrics: accuracy decreased from 0.912 to 0.903 (ΔAcc = −0.99%), Macro-F1 declined from 0.852 to 0.842, and calibration worsened (ECE 0.031 → 0.035). This pattern suggests that the Laplacian-style smoothness constraint improves generalization while mitigating overconfident errors.

**Table 4:** Results of the ablation study.

Model variant	Acc	Δ Acc	BalAcc	Macro-F1	W-F1	AUROC	AUPRC	MCC	ECE↓
**GCOA-Net (full model)**	**0.912**	–	**0.875**	**0.852**	**0.910**	**0.965**	**0.915**	**0.865**	**0.031**
Structural ablations									
(1) w/o graph regularization (λ_gr_ = 0)	0.903	–0.99%	0.861	0.842	0.898	0.960	0.907	0.852	0.035
(2) w/o GNN message passing (fusion-only)	0.891	–2.30%	0.845	0.835	0.885	0.952	0.897	0.831	0.041
Topological ablations									
(3) w/o miRNA→gene edges	0.901	–1.21%	0.858	0.846	0.896	0.959	0.906	0.849	0.034
(4) w/o Gene–CpG edges	0.898	–1.54%	0.854	0.843	0.892	0.957	0.904	0.844	0.036
(5) Randomized cross-omics edges	0.880	–3.51%	0.832	0.824	0.874	0.947	0.890	0.815	0.048

All metrics are averaged over repeated stratified five-fold cross-validation. Expected calibration error (ECE) measures the alignment between predicted probabilities and empirical accuracy; lower values indicate better calibration. ΔAcc denotes the relative accuracy drop with respect to the full model, computed as (Acc_variant_−Acc_full_)/Acc_full_. Macro-F1 denotes the unweighted mean of class-wise F1-scores, whereas W-F1 denotes the support-weighted F1-score.

A larger drop was observed when disabling heterogeneous message passing (fusion-only variant), indicating that biologically structured propagation contributes beyond attention-based fusion alone. Specifically, accuracy decreased to 0.891 (ΔAcc = −2.30%), accompanied by reduced Macro-F1 (0.835) and MCC (0.831), and a notable increase in ECE to 0.041. Together, these results imply that cross-omics neighborhood aggregation is a primary driver of both predictive performance and confidence reliability.

Topological ablations further demonstrate that both relation types provide complementary signal. Removing miRNA→gene edges reduced accuracy to 0.901 (ΔAcc = −1.21%) with mild losses in AUROC/AUPRC (0.959/0.906), whereas removing gene–CpG edges reduced accuracy to 0.898 (ΔAcc = −1.54%) and slightly worsened calibration (ECE 0.036). Notably, degree-preserving edge randomization produced the strongest degradation (accuracy 0.880; ΔAcc = −3.51%; ECE 0.048), despite preserving graph sparsity and degree statistics. This indicates that gains are not attributable to generic graph smoothing, but depend on *biologically grounded* cross-omics connectivity that aligns methylation and miRNA regulation with their gene-level targets.

### 3.4 Interpretability: cross-omics biomarkers and subtype-specific regulatory subnetworks

To interpret how GCOA-Net leverages multi-omics signals, we used established gradient-based XAI methods, specifically Saliency and Integrated Gradients, to decompose model evidence into (i) *node importance* (genes, promoter-CpG clusters, and miRNAs) and (ii) *edge importance* (gene–CpG and miRNA–gene relations). For each test sample, we computed attribution scores with respect to the predicted subtype logit and aggregated them across repeated CV folds. Node-level saliency scores were then summarized by subtype to identify consistent subtype-discriminative molecular features. To obtain compact mechanistic explanations, we additionally extracted a *subtype-specific explanatory subnetwork* by retaining the top-ranked cross-omics edges (by absolute edge attribution) until reaching a fixed sparsity budget, followed by connected-component pruning to yield a minimally sufficient, interpretable subgraph.

Table 5 summarizes the most salient subtype-discriminative signals identified by GCOA-Net across transcriptomic, epigenomic, and miRNA layers. The top-ranked genes align with canonical subtype biology: Luminal A is dominated by *ESR1* (score 0.183), Luminal B highlights proliferation via *MKI67* (0.176), HER2-enriched is centered on *ERBB2* (0.192), and Basal-like emphasizes basal cytokeratin *KRT5* (0.187). Normal-like is characterized by *SFRP1* (0.158), consistent with its distinct but weaker signal relative to major PAM50 subtypes. Importantly, each subtype's transcriptomic driver is accompanied by coherent cross-omics evidence: the highest-saliency promoter CpG clusters map to the corresponding gene loci (e.g., CpG_*ERBB2*_prom_02 score 0.148), and subtype-associated miRNAs also emerge with non-trivial attribution scores (e.g., hsa-miR-21 score 0.121 for HER2-enriched). At the cohort level, aggregated saliency highlights *GATA3* (0.169) and CpG_*GATA3*_prom_01 (0.134), suggesting a shared luminal regulatory program that may partially explain the residual Luminal A/B and Normal-like overlap observed in Section 3.2.

**Table 5:** Top subtype-discriminative cross-omics biomarkers identified by GCOA-Net attribution analysis.

	Transcriptomics		Epigenomics		miRNA regulatory	
Subtype	Top gene	Score	Top promoter-CpG	Score	Top miRNA	Score
Luminal A	*ESR1*	0.183	CpG_*ESR1*_prom_03	0.142	hsa-miR-200c	0.117
Luminal B	*MKI67*	0.176	CpG_*MKI67*_prom_01	0.131	hsa-miR-182	0.112
HER2-enriched	*ERBB2*	0.192	CpG_*ERBB2*_prom_02	0.148	hsa-miR-21	0.121
Basal-like	*KRT5*	0.187	CpG_*KRT5*_prom_01	0.139	hsa-miR-205	0.115
Normal-like	*SFRP1*	0.158	CpG_*SFRP1*_prom_02	0.126	hsa-miR-143	0.103
Global (Aggregated)	*GATA3*	0.169	CpG_*GATA3*_prom_01	0.134	hsa-miR-200a	0.110

Importance scores represent the mean absolute gradient attribution aggregated across test samples. Genes and miRNAs are italicized following standard nomenclature.

Beyond individual markers, we extracted subtype-specific explanatory subnetworks to provide compact mechanistic hypotheses. Across subtypes, the resulting graphs were sparse yet structured (Table 6), typically comprising ~103–147 nodes and 169–258 edges with a limited number of hub entities, indicating that model decisions can be explained by a small set of recurring cross-omics interactions rather than diffuse feature contributions. Moreover, the fold overlap ranged from 0.541 to 0.612, reflecting moderate stability of the identified edges under resampling and supporting that the extracted subnetworks capture reproducible cross-omics patterns.

**Table 6:** Structural characteristics of the extracted subtype-specific explanatory subnetworks.

Subtype	Nodes	Edges	Gene hubs	CpG hubs	miRNA hubs	Fold overlap
Luminal A	126	214	8	19	7	0.612
Luminal B	138	236	9	22	8	0.584
HER2-enriched	121	205	7	18	7	0.598
Basal-like	147	258	10	24	9	0.571
Normal-like	103	169	6	15	6	0.541

Explanatory subnetworks were constructed by retaining the top 1% of edges based on attribution scores. “Fold Overlap” indicates the Jaccard similarity of edges across cross-validation folds, quantifying the stability of the identified mechanisms.

**Figure 5:**
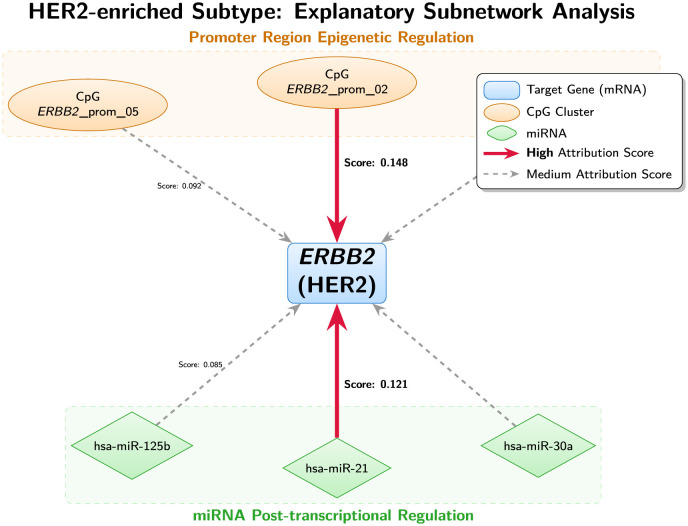
Visualization of the HER2-enriched subtype-specific regulatory subnetwork derived from GCOA-Net. The diagram illustrates the top-ranked cross-omics interactions centered around the defining oncogene *ERBB2* (HER2). Nodes represent biological entities: target genes (blue rectangles), promoter CpG clusters (orange ellipses), and miRNAs (green diamonds). Edges represent regulatory dependencies learned by the model. The thickness and color intensity of the red arrows correspond to the magnitude of gradient-based attribution scores, highlighting the most critical predictors identified by GCOA-Net.

Figure 5 illustrates the HER2-enriched explanatory subnetwork, in which *ERBB2* acts as a central hub integrating promoter-proximal epigenetic regulation (e.g., CpG_*ERBB2*_prom_02) and miRNA-mediated post-transcriptional regulation (e.g., hsa-miR-21). The concentration of high-attribution edges around the defining oncogene provides an interpretable rationale for subtype assignment and offers a clinically meaningful explanation pathway: predictions for HER2-enriched cases are supported by convergent evidence spanning transcriptional activation and multi-layer regulatory control.

From a translational perspective, these attribution-ranked markers and compact subnetworks provide a practical *prioritization map* for follow-up validation: they can be used to nominate a small set of cross-omics candidate biomarkers and regulatory links (e.g., gene–promoter CpG–miRNA triplets) for targeted assays, orthogonal confirmation (IHC/FISH for HER2 status, locus-specific methylation assays, and miRNA quantification), and hypothesis-driven studies of subtype-specific regulation, thereby bridging predictive modeling with clinically actionable molecular interpretation.

### 3.5 Robustness, calibration, and deployment-oriented analyses

**Figure 6:**
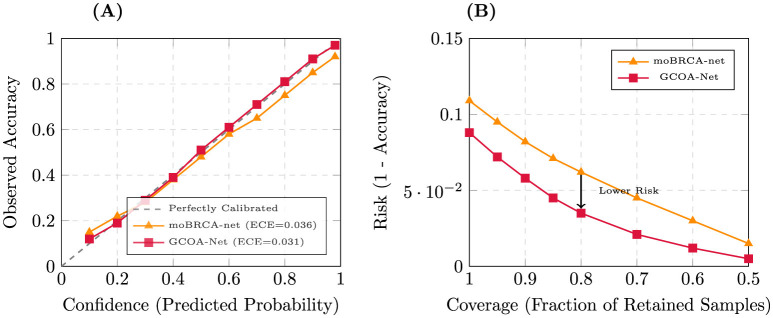
Calibration and reliability analysis of GCOA-Net vs. moBRCA-net. **(A)** Reliability diagram: this plot compares the alignment between the model's predicted confidence and its observed accuracy. **(B)** Selective prediction profile: this curve illustrates the trade-off between coverage (fraction of samples retained) and risk (error rate).

Table 7 evaluates robustness under clinically realistic missing-modality conditions, where one or more omics assays may be unavailable at deployment. Across all scenarios, GCOA-Net retained higher discrimination and better calibration than moBRCA-net. When all modalities were present, GCOA-Net achieved Acc = 0.912 and ECE = 0.031 (as detailed in Table 8 and illustrated by the reliability diagram in Figure 6A), improving over moBRCA-net (Acc = 0.891, ECE = 0.036). Under single-modality absence at inference time, GCOA-Net consistently degraded more gracefully: without mRNA, accuracy remained 0.868 vs. 0.842 for moBRCA-net (+0.026); without CpG, accuracy remained 0.885 vs. 0.858 (+0.027); and without miRNA, accuracy remained 0.901 vs. 0.875 (+0.026). These gains were mirrored by higher balanced accuracy and Macro-F1 (e.g., w/o CpG: Macro-F1 0.818 vs. 0.795) and by lower calibration error (e.g., w/o mRNA: ECE 0.046 vs. 0.052). Even in the severe data-loss setting (mRNA only), GCOA-Net maintained an advantage (Acc = 0.842 vs. 0.825; ECE = 0.052 vs. 0.057), supporting its suitability for settings where comprehensive multi-omics profiling is not feasible.

**Table 7:** Robustness analysis: performance under missing modality scenarios at inference time.

Method	Missing modality	Acc	BalAcc	Macro-F1	W-F1	ECE↓
moBRCA-net	None (full data)	0.891	0.845	0.835	0.887	0.036
**GCOA-Net**	**None (full data)**	**0.912**	**0.875**	**0.852**	**0.910**	**0.031**
moBRCA-net	w/o mRNA (CpG + miRNA)	0.842	0.795	0.776	0.810	0.052
**GCOA-Net**	**w/o mRNA (CpG + miRNA)**	**0.868**	**0.812**	**0.799**	**0.845**	**0.046**
moBRCA-net	w/o CpG (mRNA + miRNA)	0.858	0.812	0.795	0.835	0.048
**GCOA-Net**	**w/o CpG (mRNA + miRNA)**	**0.885**	**0.834**	**0.818**	**0.862**	**0.041**
moBRCA-net	w/o miRNA (mRNA + CpG)	0.875	0.830	0.821	0.868	0.041
**GCOA-Net**	**w/o miRNA (mRNA+CpG)**	**0.901**	**0.858**	**0.838**	**0.895**	**0.036**
*Severe data loss scenario:*						
moBRCA-net	mRNA Only	0.825	0.772	0.742	0.784	0.057
**GCOA-Net**	**mRNA only**	**0.842**	**0.795**	**0.760**	**0.806**	**0.052**

“w/o” denotes “without.” All metrics are averaged over repeated stratified five-fold cross-validation using out-of-fold predictions. Macro-F1 denotes the unweighted mean of class-wise F1-scores, whereas W-F1 denotes the support-weighted F1-score. ECE: Expected Calibration Error (lower is better). Within each missing-modality scenario, bold values indicate the better result between moBRCA-net and GCOA-Net for that metric. Higher values are better for Acc, BalAcc, Macro-F1, and W-F1, whereas lower values are better for ECE.

**Table 8:** Calibration metrics aggregated across out-of-fold predictions.

Method	ECE↓	Brier score↓	NLL↓
XGBoost	0.051	0.214	0.612
SNF + SVM	0.047	0.208	0.598
MOGONET	0.040	0.196	0.571
moBRCA-net	0.036	0.190	0.559
**GCOA-Net**	**0.031**	**0.182**	**0.541**

NLL, negative log-likelihood. Lower values indicate better performance for all metrics. Bold values indicate the best result in each metric column across methods. Lower values are better for all metrics.

To distinguish inference-time robustness from explicit missingness-aware training, we further compared the standard GCOA-Net with a training-time masking variant in which an entire modality was randomly masked during model fitting. As shown in Supplementary Table S6, the training-time masking variant traded a small reduction in full-data accuracy (0.912–0.906) for improved performance across all missing-modality scenarios, including w/o mRNA (0.868–0.882), w/o CpG (0.885–0.895), w/o miRNA (0.901–0.905), and the severe-loss setting (0.842–0.865). This pattern indicates that the robustness of GCOA-Net under missing modalities is not limited to *post-hoc* masking at test time, but can be further strengthened through missingness-aware training.

Finally, we refined the selective prediction protocol by choosing abstention thresholds on the validation split rather than on the test data. A representative validation-set accuracy–coverage curve is shown in Supplementary Figure S1, where target coverage rates of 90%, 80%, and 70% determine fixed operating thresholds that are subsequently applied unchanged to the held-out test fold. Under this protocol, GCOA-Net continued to show a more favorable coverage–risk trade-off than moBRCA-net in Figure 6B, supporting its use in settings where uncertain cases may be deferred for additional review or testing.

Because the primary evaluation of GCOA-Net was conducted within TCGA-BRCA, we additionally performed a stronger site-stratified internal generalization analysis based on held-out Tissue Source Sites. As shown in Supplementary Table S5, GCOA-Net remained the best-performing model among the strongest deep comparators under this site-held-out protocol, achieving the highest mean Accuracy (0.903), mean Macro-F1 (0.841), and mean AUROC (0.956). Relative to moBRCA-net, the best-performing baseline in the main repeated cross-validation analysis, GCOA-Net also retained stronger worst-site performance, with worst-site Accuracy improving from 0.841 to 0.872 and worst-site Macro-F1 improving from 0.785 to 0.815. These results suggest that the gains of GCOA-Net are not solely driven by random resampling within TCGA, but remain evident when evaluation is conducted across major source-site partitions.

## 4 Discussion

### 4.1 Clinical motivation and principal findings

Intrinsic subtypes of breast cancer capture clinically actionable biology and remain a cornerstone for treatment stratification, particularly for distinguishing endocrine-responsive Luminal tumors, HER2-driven disease, and Basal-like phenotypes that often require intensified systemic therapy (1–3, 41). Using the TCGA-BRCA multi-omics cohort (4), our results show that **GCOA-Net** achieves consistent improvements over classical early-fusion baselines and representative multi-omics integration frameworks, with the strongest margins over graph-based and attention-based deep competitors. Beyond overall accuracy gains, the subtype-wise analysis indicates that the improvements are not uniform: the model yields the most practical benefit where subtype assignment often drives distinct clinical pathways (e.g., HER2-enriched and Basal-like), while residual ambiguity persists along biologically proximate boundaries (e.g., Luminal A/Luminal B and Normal-like/Luminal A), a pattern frequently observed in expression-based taxonomies and large cohort annotations (3, 4).

### 4.2 Why heterogeneous cross-omics graph modeling improves subtype discrimination

A key methodological insight is that *how* omics layers are integrated matters as much as *whether* they are integrated. Classical early-fusion models (e.g., RF, XGBoost) can be competitive but typically treat features as an unstructured vector, relying on generic interactions learned from limited samples (29, 30). By contrast, network-based integration methods (SNF) and latent-component approaches (DIABLO) encode cross-modal structure more explicitly, but still do not directly represent mechanistic regulatory relations at the entity level (5, 32). Recent graph-based patient similarity methods (e.g., MOGONET, SUPREME) further demonstrate that graph learning can strengthen multi-omics prediction and support biomarker discovery (11, 12). GCOA-Net advances this direction by constructing a *heterogeneous molecular graph* that links genes, promoter-proximal CpG clusters, and miRNAs through biologically grounded relations, allowing message passing to operate on interpretable regulatory neighborhoods rather than purely sample-to-sample similarity.

The ablation findings are consistent with this mechanistic hypothesis: removing GNN message passing or randomizing cross-omics edges yields the largest degradation, indicating that performance gains depend on structured cross-omics connectivity rather than generic smoothing. Moreover, the benefit of graph regularization aligns conceptually with manifold regularization–encouraging representations to vary smoothly along plausible biological relations and thereby stabilizing learning under high-dimensional, noisy molecular measurements (22). At the architectural level, heterogeneous relational modeling is also supported by broader relational GNN theory, where typed edges enable learning different transformation rules for distinct biological relations (e.g., CpG → gene vs. miRNA→gene) (21).

### 4.3 Translational interpretability: biomarker consistency and subtype-specific regulatory neighborhoods

For clinical translation, predictive performance alone is insufficient: models should generate *auditable evidence* and connect predictions to known biology and potential intervention points. Our attribution analysis provides two complementary explanation layers: (i) node saliency highlights subtype-discriminative entities across omics, and (ii) edge attribution surfaces cross-omics interactions that the model uses to support subtype decisions. Notably, the highest-saliency hubs align with canonical subtype biology: ESR1-related signals for Luminal A, proliferation-associated markers for Luminal B, ERBB2-centered evidence for HER2-enriched, and basal cytokeratin programs for Basal-like tumors–patterns repeatedly observed in intrinsic subtype studies and TCGA molecular portraits (1–4). From a translational standpoint, this alignment is important because it indicates that the model does not rely on spurious correlates; instead, it re-discovers clinically meaningful axes of variation from multi-omics data.

The HER2-enriched explanatory subnetwork (Figure 5) illustrates how cross-omics explanations can be used as a *hypothesis generator* for clinically testable mechanisms. Edges linking promoter CpG clusters to ERBB2 provide an epigenetic route by which regulatory state may modulate the HER2 program, while miRNA→gene relations suggest post-transcriptional control points. Because miRNA targeting is grounded in established resources (e.g., TargetScan), the extracted miRNA–gene interactions can be mapped to prior knowledge to prioritize candidates for orthogonal validation (20). At the cohort level, the observed residual confusions (e.g., Luminal/Normal-like overlap) are clinically familiar in TCGA-style datasets, where tumor purity, stromal admixture, and expression proximity can blur boundaries; importantly, such ambiguity underscores the value of the interpretability layer as a safeguard for downstream review and motivates the following deployment-oriented analyses (3, 4).

For clinical translation, predictive performance alone is insufficient: models should generate *auditable evidence* and connect predictions to known biology and potential intervention points. Our interpretability analysis is grounded in established XAI methods rather than *ad hoc* feature scoring. Specifically, node-level explanations were derived using Saliency and Integrated Gradients, whereas edge-level explanations were obtained through differentiable edge gating within the graph model. These complementary explanation layers allow us to identify subtype-discriminative entities across omics and to surface cross-omics interactions that the model uses to support subtype decisions.

### 4.4 Deployment-oriented reliability: calibration and selective prediction as clinical safety levers

For deployment, subtype prediction is frequently used in pipelines that culminate in therapy recommendation or trial eligibility, making probabilistic reliability a practical requirement. Our calibration analysis shows that GCOA-Net improves probability calibration over strong deep baselines, which is consistent with the broader literature documenting that modern neural models can be miscalibrated and that reliability diagrams/ECE provide actionable diagnostics (18, 34, 35). Clinically, better calibration means that a “high-confidence HER2-enriched” output is more likely to correspond to true correctness, enabling triage workflows in which uncertain cases are escalated for additional assays or expert review.

Selective prediction further operationalizes this principle by explicitly trading coverage for risk using a reject option (39, 42). In a realistic multi-omics setting, where assay availability, batch effects, and turnaround time vary, such a coverage–risk curve can support a tiered strategy: (i) accept high-confidence calls for rapid stratification; (ii) defer borderline cases for confirmatory testing (e.g., targeted panels, IHC/FISH for HER2 per guideline), and (iii) document the evidence subnetwork to support molecular tumor board interpretation (41, 43). This connects naturally to contemporary recommendations that explainable clinical AI should be designed around end-user needs and decision context, rather than explanations as an afterthought (19, 43).

### 4.5 Positioning within the multi-omics ecosystem and forward-looking clinical integration

From a broader perspective, GCOA-Net is positioned at the intersection of multi-omics integration, graph learning, and clinically oriented reliability analysis. Its contribution differs from prior methods at several levels. At the level of problem formulation, many multi-omics studies focus primarily on discrimination performance, whereas our formulation emphasizes subtype-oriented prediction together with interpretability, calibration, selective prediction, and robustness to missing modalities. This distinction is especially important in breast cancer subtyping, where model outputs may influence downstream review, therapy stratification, and further molecular confirmation (3, 4, 41).

At the architectural level, GCOA-Net differs from several major streams of prior work. Relative to latent-factor and representation-learning approaches such as MOFA+, our framework does not aim primarily to summarize shared and modality-specific variation in a low-dimensional latent space, but instead explicitly represents biologically grounded cross-omics relations among molecular entities (7). Relative to classical early-fusion and integration frameworks, such as SNF and DIABLO, GCOA-Net does not treat multi-omics structure as either simple feature concatenation or sample-level alignment alone (5, 32). Relative to patient-similarity graph approaches such as MOGONET and SUPREME, the proposed graph is constructed at the molecular-entity level rather than at the patient level, allowing message passing to operate over biologically interpretable regulatory neighborhoods rather than over sample-to-sample similarity graphs (11, 12). More recent studies such as MVGNN, MOGAT, GAIN-BRCA, MO-GCAN, and MOFNet further illustrate the rapid development of graph-based and attention-based multi-omics subtype models, but these methods still mainly rely on patient-level similarity structures, transformed feature fusion, or label-space integration rather than an explicitly heterogeneous regulatory graph over genes, CpG clusters, and miRNAs (13–17). In this sense, the present model extends recent graph-based multi-omics learning by shifting the graph inductive bias from inter-patient resemblance to mechanistically motivated cross-omics connectivity (8, 44). At the level of learning strategy, the present study goes beyond attention-based fusion alone. Compared with breast cancer-oriented deep baselines such as moBRCA-net (23), our model adds graph regularization that encourages local consistency along biologically plausible cross-omics edges and constrains information propagation using explicit molecular priors. The ablation results support the value of this design, showing that both heterogeneous message passing and graph regularization contribute to improved discrimination and more stable performance under missing-modality and site-held-out evaluation settings.

The present work also differs from much of the recent multi-omics prediction literature in the scope of empirical evaluation. Rather than limiting comparison to average classification performance, we additionally examine subtype-wise error structure, attribution-based biomarker discovery, explanatory subnetworks, calibration, selective prediction, corrected repeated cross-validation inference, and stronger site-stratified within-cohort generalization. In this sense, the contribution of GCOA-Net is not only a new architecture, but also a more deployment-aware evaluation perspective for interpretable multi-omics subtype prediction (8, 9).

Finally, our positioning also differs in how interpretability is operationalized. We do not treat explanation as a purely auxiliary visualization layer, but instead analyze both node-level and edge-level attributions, subtype-wise marker consistency, and sparse explanatory subnetworks. These choices are aligned with established gradient-based XAI methods in deep learning and with emerging approaches for explaining graph-based predictions (37, 38, 45). In parallel, recent deep generative approaches for multi-omics, such as multiDGD, highlight a complementary direction by learning coherent latent structure that may support simulation, representation transfer, and data augmentation (46, 47). Future work may therefore explore hybrid systems that combine generative modeling with biologically grounded heterogeneous graphs for breast cancer molecular stratification.

## 5 Conclusion

In this study, we presented GCOA-Net, a graph-regularized cross-omics attention network for interpretable breast cancer intrinsic subtype classification from transcriptomic, promoter-proximal DNA methylation, and miRNA data. By embedding biologically grounded cross-omics relations into a heterogeneous graph and coupling relation-aware message passing with multi-level attention, GCOA-Net achieved consistently improved discrimination over classical, integration-based, and deep multi-omics baselines, while yielding stable subtype-specific explanations in the form of salient biomarkers and compact regulatory subnetworks.

Beyond improved discrimination, our results indicate that GCOA-Net remains comparatively robust under missing-modality conditions and under a stricter site-held-out internal generalization setting, while also producing better-calibrated probabilities and more stable subtype-specific explanations. At the same time, all current evidence remains internal to TCGA-BRCA. Accordingly, the present findings should be viewed as support for future validation in independent multi-omics cohorts rather than as definitive evidence of broader clinical transportability.

A further limitation is that all model assessment in this study remains internal to TCGA-BRCA. To strengthen the generalization analysis, we added a site-held-out evaluation across the largest Tissue Source Site groups, which provides a more demanding within-cohort robustness test than ordinary random cross-validation. Even so, this analysis does not replace validation in an independent external multi-omics cohort. Future work should therefore examine whether the observed gains of GCOA-Net are preserved across cohorts generated under different sampling, processing, and profiling conditions.

## Data Availability

The datasets analyzed in this study are publicly available from the Cancer Genome Atlas Breast Invasive Carcinoma (TCGA-BRCA) project via the Genomic Data Commons Data Portal: https://portal.gdc.cancer.gov/projects/TCGA-BRCA.
