# Supplementary material for: GCOA-Net: a graph-regularized cross-omics attention network for interpretable breast cancer molecular subtype classification
Source: Front Med (Lausanne). 2026 Apr 10;13:1790437. doi: 10.3389/fmed.2026.1790437 (PMC13105865; doi:10.3389/fmed.2026.1790437)
Supplement: Supplementary file 1 [file Data_Sheet_1.pdf]

Supplementary Materials

Supplementary Table S1. Key parameters and harmonization rules for heterogeneous prior-graph construction.

Item	Specification
Genome build	GRCh38 (hg38)
Probe coordinate source	GDC liftover annotation for HM450
Gene annotation source	GENCODE v36
Gene unit	Official HGNC gene symbol
Transcript handling	Union of transcription start sites
Promoter definition	2000 bp upstream to 500 bp downstream of TSS
Gene–CpG mapping rule	Many-to-many
CpG input unit	Illumina HumanMethylation450 probes
CpG clustering rule	Genomic proximity within 500 bp
Minimum cluster size	At least 2 probes
Cluster aggregation rule	Arithmetic mean of beta values
Probe missingness filter	Remove probes with more than 10% missing samples
miRNA target database	TargetScan Human
Database version	Version 8.0
Interaction type retained	Conserved target sites only
Filtering threshold	Cumulative weighted context++ score ≤ -0.2
Gene identifier harmonization	biomaRt-based mapping
miRNA naming harmonization	Mature miRNA names based on miRBase v22
Unmatched entity rule	Excluded
Duplicate feature-mapping rule	Highest cross-sample variance retained
Edge deduplication rule	Collapsed to unique node pairs
Graph usage across resampling	Fixed before cross-validation

Supplementary Table S2. Node and edge accounting during heterogeneous graph assembly.

Stage	Description	Gene nodes	CpG-cluster nodes	miRNA nodes	Gene–CpG edges	miRNA→gene edges
1	Raw features	60,483	485,577 (probes)	1,881	–	–
2	After preprocessing and clustering	20,400	19,977	1,597	–	–
3	Initial gene–CpG overlaps	20,400	19,977	–	45,610	–
4	Promoter-filtered gene–CpG links	20,400	19,977	–	34,228	–
5	Raw miRNA–target pairs	–	–	1,720	–	985,420
6	Score-filtered miRNA–target pairs	–	–	1,650	–	365,112
7	After TCGA intersection and ID cleaning	20,400	19,977	1,597	34,228	348,387
8	Final graph	20,400	19,977	1,597	34,228	348,387

Supplementary Table S3. Baseline implementation sources and hyperparameter optimization details.

Model	Implementation	Software / ver.	Tuning strategy	Key hyperparameters	Stopping rule
SVM (RBF)	Standard pkg	scikit-learn v1.4.2	Grid search (5-fold inner CV)	$C \in \{10^{-3}, \ldots, 10^{3}\}$; $\gamma \in \{10^{-4}, \ldots, 10^{0}\}$	Tol. $< 10^{-3}$
Random Forest	Standard pkg	scikit-learn v1.4.2	Random search (5-fold inner CV)	$n\_est \in [100, 1000]$; $depth \in [5, 20]$; $split \in [2, 10]$	Inner-CV perf.
XGBoost	Standard pkg	xgboost v2.0.3	Grid search (5-fold inner CV)	$depth \in \{3, 6, 9\}$; $lr \in \{0.01, 0.1\}$; $sub \in \{0.8, 1.0\}$	Early stop ($p = 50$)
Elastic Net	Standard pkg	scikit-learn v1.4.2	Grid search (5-fold inner CV)	$\alpha \in [10^{-4}, 10^{1}]$; $l1\_ratio \in [0.1, 0.9]$	Tol. $< 10^{-4}$
SNF + SVM	Package-based	SNFtool (R v2.3.1)	Rec. settings + inner-CV SVM	$K \in \{20, 30\}$; $\mu \in \{0.5, 0.8\}$; C, γ tuned by inner CV	Fixed iterations
DIABLO	Package-based	mixOmics v6.24.0	M-fold inner CV	Comps $\in \{2, \ldots, 5\}$; dist $\in$ {max.dist, centroids.dist}	BER minim.
DeepMO-style	Reimplementation	PyTorch	Grid search (inner)	$lr \in \{10^{-3}, 10^{-4}\}$; $hidden \in \{128, 256\}$; $drop \in [0.2, 0.5]$	Early stop ($p = 30$)
MOGONET	Official (adapted)	PyTorch (official)	Random search (inner)	$lr \in \{10^{-3}, 10^{-4}\}$; $hidden \in \{128, 256\}$; $drop \in [0.2, 0.5]$	Early stop ($p = 20$)
SUPREME	Official (adapted)	PyTorch (official)	Rec. settings	$dim = 64$; $layers = 2$; $lr = 0.001$; $wd = 5 \times 10^{-4}$	Fixed 200 epochs
moBRCA-net	Official (adapted)	PyTorch (official)	Grid search (inner)	$lr \in \{10^{-3}, 5 \cdot 10^{-4}\}$; $bs \in \{32, 64\}$; $smooth = 0.1$	Early stop ($p = 30$)
GCOA-Net	Custom	PyTorch Geometric	Grid search (inner)	$lr \in \{10^{-3}, 5 \cdot 10^{-4}\}$; $\lambda_{gr} \in \{0.1, 0.5, 1.0\}$; $ch \in \{64, 128\}$	Early stop ($p = 30$)

Note: Standard pkg: standard scikit-learn and package implementation. Official: official repository code with minor data-interface adjustments. Reimplementation: model implemented based on the original methodological description. p: patience for early stopping. lr: learning rate.

Supplementary Table S4. Corrected repeated cross-validation inference for GCOA-Net versus the three strongest deep learning baselines.

Baseline	Metric	Mean difference (GCOA-Net − Baseline)	Corrected 95% CI	Two-sided corrected P
**moBRCA-net**	Accuracy	+0.021	[0.003, 0.039]	0.026
	Macro-F1	+0.017	[0.001, 0.033]	0.038
	AUROC	+0.013	[0.002, 0.024]	0.020
	AUPRC	+0.030	[0.012, 0.048]	0.008
**SUPREME**	Accuracy	+0.049	[0.025, 0.073]	<0.001
	Macro-F1	+0.035	[0.016, 0.054]	0.002
	AUROC	+0.017	[0.005, 0.029]	0.006
	AUPRC	+0.037	[0.018, 0.056]	<0.001
**MOGONET**	Accuracy	+0.054	[0.029, 0.079]	<0.001
	Macro-F1	+0.041	[0.020, 0.062]	<0.001
	AUROC	+0.021	[0.008, 0.034]	0.003
	AUPRC	+0.043	[0.024, 0.062]	<0.001

Note: Mean differences are calculated as (GCOA-Net − Baseline). Confidence intervals and P-values were computed using the Nadeau–Bengio corrected repeated cross-validation test, adjusting for dependence across the 25 evaluation folds (5 repeats × 5 folds).

Supplementary Table S5. Site-held-out internal generalization performance.

Model	Paradigm	Mean Acc.	Mean M-F1	Mean AUROC	Worst Acc.	Worst M-F1
moBRCA-net	Attention Fusion	0.882	0.824	0.941	0.841	0.785
SUPREME	Patient Graph	0.851	0.798	0.932	0.805	0.742
MOGONET	Patient Graph	0.845	0.789	0.928	0.792	0.730
**GCOA-Net**	**Mol. Graph Reg.**	**0.903**	**0.841**	**0.956**	**0.872**	**0.815**

Note: Metrics were calculated from site-held-out evaluation across the five Tissue Source Site groups in TCGA-BRCA (BH, A2, AR, E2, and AC). In each run, one site was held out for testing and the remaining selected sites were used for model fitting. M-F1: Macro-F1. Worst-site metrics denote the lowest performance observed among the held-out sites.

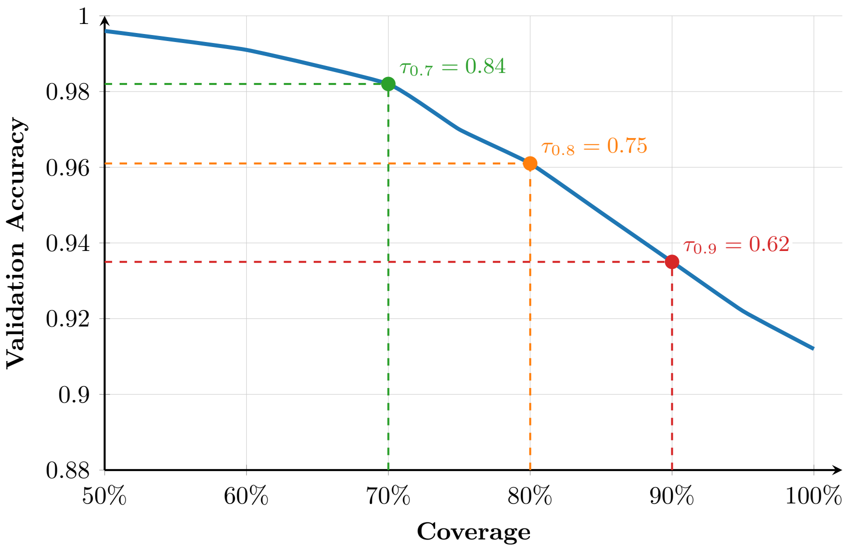

Supplementary Figure S1. Validation-based threshold selection for the selective prediction protocol. The plot illustrates the accuracy–coverage trade-off curve calculated exclusively on the internal validation split. Vertical dashed lines indicate pre-defined target coverage rates of 90%, 80%, and 70%. The intersections with the curve define the corresponding confidence thresholds ($\tau_{0.9} = 0.62$, $\tau_{0.8} = 0.75$, $\tau_{0.7} = 0.84$).

Supplementary Table S6. Effect of training-time masking on missing-modality robustness of GCOA-Net.

**Model Variant**	**Full Data Accuracy**	**Inference-time Missingness Scenarios (Accuracy)**			
		**w/o mRNA**	**w/o CpG**	**w/o miRNA**	**Severe Loss (mRNA only)**
**GCOA-Net** (Standard)	**0.912**	0.868	0.885	0.901	0.842
**GCOA-Net** (Training-time masking)	0.906	**0.882**	**0.895**	**0.905**	**0.865**

Note: Values represent mean accuracy across repeated 5-fold cross-validation. The standard variant matches the primary missing-modality analysis reported in the main text. "w/o" indicates the complete absence of the specified modality during test-time inference. "Severe Loss (mRNA only)" denotes the scenario in which both CpG and miRNA data are missing at inference time.
